# Annotations

**Published:** 1905-09-02

**Authors:** 


### ANNOTATIONS.

### Hospital Treatment for Mental Affections.

The annual report issued by Mr. John Carswell, certifying medical officer in lunacy cases for the Glasgow Parish Council, is always a document of interest and it does not on the present occasion fall behind the records of previous years. For some time Mr. Carswell has urged that under appropriate hospital treatment many cases commonly regarded as certifiable can be saved from the necessity of confinement in an asylum, and by the help of the authorities he has been able to put this policy to a practical test. The figures he now submits certainly afford strong support to his contention. Thus of 1,027 cases reported to the inspector of poor as lunatics, 449 were certified as insane and were at once removed to an asylum. Of the remainder, 472 were after examination considered to be suitable for treatment in the special hospital devoted to the care of early and transient mental cases. Subsequently, however, 134 of these were recognised as cases of lunacy and were in due course removed to an asylum. Thus there were left 338 patients out of the original 1,027 who were subjected solely to special hospital treatment, and of these 213 recovered and were discharged, whilst an additional 95 were so far "relieved" that they were either sent home or removed to the wards of a general hospital. Such results are very impressive and indicate that the procedure adopted is greatly to the advantage of both patients and the public. They have been attained by careful examination of all cases reported to the inspectors under the Poor Law Act with a view to determine whether the mental condition undeniably demands certification and detention or whether, under influences that can be secured in a special hospital, there is a reasonable prospect that it will subside and disappear. Mr. Carswell objects to the special hospital being described as an "observation ward," as this suggests it is a place for the purpose of diagnosis. He urges, on the contrary, that every attempt should be made to make a diagnosis beforehand, so that the undoubted lunatic may at once be sent to an asylum, and the hospital be reserved for cases which may reasonably be expected to recover.

### Socialism and Common Sense.

Constance May Gammon, aged four months, has fallen a victim to her father's regard for the franchise. Gammon was out of work and his family were starving. A doctor called in to see the child who has now died, recommended the mother to apply for parish relief, but she did not dare to do so because her husband, who is a Socialist, had a strong objection to being disfranchised. The inspector of the National Society for the Prevention of Cruelty to Children gave evidence that she had complained to him that her husband had given a loaf to a "comrade" when his own children were in want, and a neighbour quoted a remark of the mother's which seemed to indicate that Gammon had bought flowers when bread was more needed. The whole incident seems to show a lack of balance in the man's mind which is perhaps the best excuse that can be offered for his conduct. But the claim that a man should not lose his vote by accepting parish relief is now frequently made, and yet the law which gives a man a choice between being a contributor and a voter or the recipient of relief and a non-voter is a very reasonable one. If we understand their principles aright, Socialists would, if they had the power, be the first to penalise non-producers, and a non-producer in rags is not more a contributor to the national wealth than a non-producer in purple and fine linen. It may be said that Gammon made his choice, and kept his vote at the cost of starving his child. One would hesitate to deprive any man of his freedom of choice between bread and principle, but it is much to be wished that Socialists of Gammon's order would at least refrain from marriage and fatherhood, so that no one but themselves might suffer from their ideas.

### A Pocket Pharmacopœia.

The British Pharmacopœia enjoys in abundant measure the amending zeal and attention of a never-resting and constantly-recruited body of critics. No sooner does a new edition appear than it is subjected to a thousand scrutinising minds, and its sins of omission and of commission are announced without excess of charity. This process is continued with little or no slackening of energy until in the course of time a further issue takes place, when, once again, the critical discipline starts with renewed vigour. No one can doubt that from all this the official volume gains much, and without question numerous successive improvements in detail are effected by the activity of its self-constituted critics. With the authorities rests the responsibility of separating the grain from the chaff, and their judgment, though not infallible, can claim a large share of practical success. One of the latest critics, however, is the advocate of a policy of the "root and branch" order, for his ambition is so to reduce the size of the Pharmacopœia that it may be found "in the pocket of every medical student and qualified practitioner." At the same time, and with the view, presumably, to secure this happy arrangement, he would have attached to each of the official remedies "a therapeutical and prescribing note." What is true of books in general doubtless applies to the Pharmacopœia in particular, namely, that so long as the volume contains what is essential the smaller it is the better. Therefore, if the descriptions of the official remedies can be condensed without loss, by all means let this be done. But the Pharmacopœia does not exist for the "pocket" of the medical student and practitioner, and, however much it may be condensed, it will not, we think, find its way to this locality. The principal interest of the medical profession in the official list is that it shall contain an authoritative definition of the substances more or less generally used in physicians' prescriptions, so that these documents may receive a uniform interpretation from every pharmacist. Certainly there is the greatest possible objection to official directions on prescribing and therapeutics. The proposal to include these is not new, and it indicates a radical misapprehension of the purpose of the Pharmacopœia.

